# An unusual “venous circle” of the internal mammary vein encountered during microvascular anastomosis and implications for practice

**DOI:** 10.1080/23320885.2020.1754129

**Published:** 2020-04-22

**Authors:** S. Samaras, M. A. McKelvie, G. Oni, C. M. Malata

**Affiliations:** a Department of Plastic & Reconstructive Surgery, Cambridge University Hospitals NHS Foundation Trust, Cambridge, UK; b Department of Plastic & Reconstructive Surgery, 401 Military Hospital of Athens, Athens, Greece; c Anglia Ruskin School of Medicine, Anglia Ruskin University, Cambridge and Chelmsford, UK; d Cambridge Breast Unit, Cambridge University Hospitals NHS Foundation Trust, Cambridge, UK

**Keywords:** Breast reconstruction, microsurgery, mammary arteries, regional anatomy, free tissue flaps

## Abstract

The internal mammary vessels are commonly used for anastomosis in breast reconstruction. The anatomy when using the 2nd ICS has been shown to be predictable and hence preferentially used by the senior author. We present an unusual case of internal mammary vein bifurcation and immediate confluence forming a ‘venous circle’.

## Introduction

The internal mammary vessels are the recipient vessels of choice worldwide for microvascular anastomosis in breast reconstruction [1–5]. Total rib-preserving vessel preparation is now well established and it involves exposing the vessels in either the 2nd or the 3rd intercostal space (ICS). Internal mammary vein (IMV) anatomy has been widely studied by Rohrich and Arnez, and is highly variable [2,3,6,7]. We proved that the pertinent vessel anatomy of the 2nd ICS is much more predictable with a larger space and a single vein in more than 80% of the cases hence it is our preferred technique [8]. We present an unusual case of internal mammary vein bifurcation and immediate confluence forming a ‘venous circle’ encountered during microvascular dissection in the second ICS and discuss the surgical implications.

## Case report

**Figure 1.**
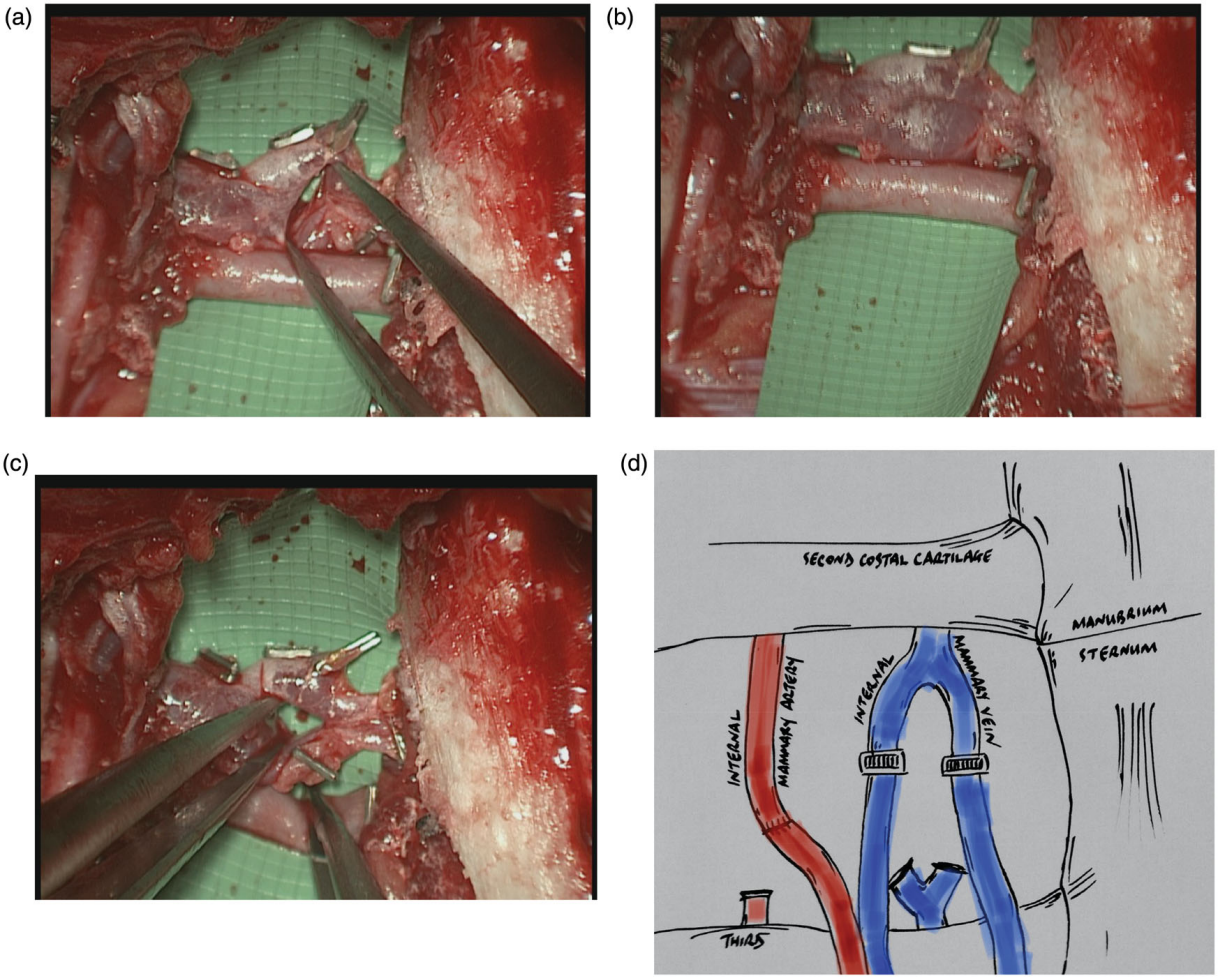
(a–d) Intraoperative photographs and graphic illustration of the left 2nd intercostal space demonstrating the “venous ring” of the internal mammary vein, medial to the artery.

A 40-year old lady was undergoing bilateral risk-reducing mastectomies and immediate breast reconstruction using bilateral deep inferior epigastric artery perforator (DIEP) flaps. A rib-preservation dissection technique [8–10] in the right second intercostal space (23 mm wide), was used to expose the recipient internal mammary vessels, with the vein found in a typical anatomical arrangement, running medial to the artery. The vein, exposed between the second and third costal cartilages, exhibited unusual anatomy in the form of the single vein forming a ‘bifurcation’, quickly followed cranially by the two vessels converging back into a single vessel, thus creating a venous ‘ring’ (Figure 1(a–c)). The corresponding artery showed typical anatomy, being lateral to the vein, existing as a single vessel throughout the intercostal space [8]. Standard vessel anatomy was also displayed on the contralateral side for both artery and vein. The recipient mammary vein was divided transversely across both bifurcating limbs and then anastomosed end-to-end to the two deep inferior epigastric veins using venous couplers (size 3 mm and 2.5 mm – Synovis GEM Microvascular Anastomotic COUPLER Device^TM^. The flap transfers were successful.

## Discussion

Internal mammary vein anatomy has been widely studied and is highly variable both in terms of number and patterns of division/confluence [2,3,6,7]. Four patterns of IMV anatomy were described by Arnez classifying the relationship of the vein to the internal mammary artery (IMA) [3]. In the most common arrangements (Arnez Type I & II) the vein is found running medial to the artery, making up 95% of cases. In Type I subjects, the vein is formed by the confluence of the two venae commitantes of the IMA, at the level of the third costal cartilage, continuing cranially as a single vein in 85% of cases [3]. These findings are consistent with our in-vivo study of the IM vessels anatomy of the 2nd and 3rd ICS. We identified a single vein cranially to the 3rd rib in more than 80% of the cases which was almost always (92%) lying medial to the artery [8]. The rib-preservation method for vessel exposure, first described by Parrett et al. in 2008 [9] and subsequently adopted and refined by the senior author [10], is widely seen as an easy, safe and reliable method of IMV exposure. Its advantages over the traditional rib-sacrificing method – whereby the second and/or third rib is resected parasternally to facilitate exposure of the recipient vessels – include faster recovery times, reduced analgesic requirements post-operatively and better preservation of normal chest wall contour [11–14].

This particular case of unusual venous anatomy raises specific issues related to microvascular anastomosis including problems with finding an ideal site of anastomosis and concerns with potentially creating a site of turbulent flow if this variant is not dealt with correctly. A decision was made during the operation to preserve and incorporate the natural confluence of the two birfurcating veins for a number of reasons.

Firstly, to exclude the venous anomaly by dividing the single IMV cranial to the ring would leave the length of recipient vessel prohibitively short for the coupler mechanism for the anastomosis, potentially risking its integrity and avulsion of the repair or IMV with excessive tension. Conversely, completely preserving the ring by dividing caudal to the bifurcation would require rib sacrifice in order to adequately expose the caudal end of the divided vein; this therefore increases the risk of the adverse outcomes associated with this method – particularly pertinent to a bilateral reconstruction case where symmetry is important and by having differing methods of vessel exposure on each side this would potentially risk asymmetrical chest wall contour [11].

Secondly, modifying the ring by ligating, excluding and excising one limb of the bifurcation in order to create a single continuous vein onto which a single donor vessel can be anastomosed potentially raises the risk of creating additional sites of turbulence at the points of ligation in the remaining limb. This would increase the risk of thrombogenesis or luminal narrowing and thus the chance of venous outflow problems. Lastly and perhaps most importantly, anastomosing two antegrade veins can be seen as advantageous as there are reports of retrograde anastomoses having an increased thrombotic risk and decreased flow rate compared to the antegrade [15]. Given the aforementioned reasons, the authors propose that by anastomosing within the ring, respecting the observed anatomy and the natural contour of the converging veins, this would preserve the integrity of the tunica intima promoting normal laminar blood flow.

In summary, we present a variation in internal mammary vein anatomy which we believe has not previously been described in the literature. Not only does this case report add to the body of work on the internal mammary vessel anatomy, but it will also guide microvascular reconstructive surgeons who are faced with unexpected anatomical variations with their intra-operative decision-making and ultimately successful flap transfer.
